# Autophagy promotes metastasis and glycolysis by upregulating MCT1 expression and Wnt/β-catenin signaling pathway activation in hepatocellular carcinoma cells

**DOI:** 10.1186/s13046-018-0673-y

**Published:** 2018-01-19

**Authors:** Qing Fan, Liang Yang, Xiaodong Zhang, Yingbo Ma, Yan Li, Lei Dong, Zhihong Zong, Xiangdong Hua, Dongming Su, Hangyu Li, Jingang Liu

**Affiliations:** 1 Department of General Surgery, The Fourth Affiliated Hospital, China Medical University, Shenyang, 110032 China; 2 Tumour Angiogenesis and Microenvironment Laboratory (TAML), Department of Oncology, The First Affiliated Hospital, Jinzhou Medical University, Jinzhou, 121000 China; 3 Departments of Laparoscopic Surgery, The First Affiliated Hospital, Dalian Medical University, Dalian, Liaoning 116001 China; 4 Department of Biochemistry and Molecular Biology, College of Basic Medicine, China Medical University, Shenyang, 100013 China; 5 Department of Hepatobiliary Surgery, Liaoning Cancer Hospital and Institute, Shenyang, 110042 China; 6 Center of Cellular therapy, the Second Affiliated Hospital, Nanjing Medical University, Nanjing, 210011 China

**Keywords:** Autophagy, Glycolysis, MCT1, Wnt/β-catenin signaling

## Abstract

**Background:**

Autophagy is a dynamic physiological process that can generate energy and nutrients for cell survival during stress. Autophagy can regulate the migration and invasive ability in cancer cells. However, the connection between autophagy and metabolism is unclear. Monocarboxylate transporter 1 (MCT1) plays an important role in lactic acid transport and H^+^ clearance in cancer cells, and Wnt/β-catenin signaling can increase cancer cell glycolysis. We investigated whether autophagy promotes glycolysis in hepatocellular carcinoma (HCC) cells by activating the Wnt/β-catenin signaling pathway, accompanied by MCT1 upregulation.

**Methods:**

Autophagic activity was evaluated using western blotting, immunoblotting, and transmission electron microscopy. The underlying mechanisms of autophagy activation on HCC cell glycolysis were studied via western blotting, and Transwell, lactate, and glucose assays. MCT1 expression was detected using quantitative reverse transcription–PCR (real-time PCR), western blotting, and immunostaining of HCC tissues and the paired adjacent tissues.

**Results:**

Autophagy promoted HCC cell glycolysis accompanied by MCT1 upregulation. Wnt/β-catenin signaling pathway activation mediated the effect of autophagy on HCC cell glycolysis. β-Catenin downregulation inhibited the autophagy-induced glycolysis in HCC cells, and reduced MCT1 expression in the HCC cells. MCT1 was highly expressed in HCC tissues, and high MCT1 expression correlated positively with the expression of microtubule-associated protein light chain 3 (LC3).

**Conclusion:**

Activation of autophagy can promote metastasis and glycolysis in HCC cells, and autophagy induces MCT1 expression by activating Wnt/β-catenin signaling. Our study describes the connection between autophagy and glucose metabolism in HCC cells and may provide a potential therapeutic target for HCC treatment.

**Electronic supplementary material:**

The online version of this article (10.1186/s13046-018-0673-y) contains supplementary material, which is available to authorized users.

## Background

Hepatocellular carcinoma (HCC) is one of the most malignant tumors worldwide [1]. Diagnosing HCC is not difficult; however, HCC treatment does not yield the expected effects. Hence, studying the key molecular mechanism of HCC development is a high priority for discovering an effective treatment.

HCC development is accompanied by cell energy metabolism that changes from oxidative phosphorylation to aerobic glycolysis, and which is termed the Warburg effect [2]. This metabolic pathway transformation not only ensures adequate energy supply to tumor cells, but also provides sufficient materials for rapid proliferation. A gas chromatography–mass spectrometry study of the metabonomics of 31 HCC tissues and paracancerous tissues showed that HCC tissues had twice the metabolism rate for glucose, glycerol 3-phosphoric acid, malic acid, alanine, inositol, and linoleic acid compared to the paracancerous tissue, and that the glycolysis capacity was four times that of oxidative phosphorylation [3]. Therefore, the rapid growth and cell activity of HCC are closely related to its glycolytic state. The characteristics of rapid growth and proliferation imply that HCC cells require much energy and sufficient material for synthesizing biological macromolecules. However, the formation of new blood vessels cannot provide the energy required for HCC cell growth, which leads to HCC cells often growing in a hypoxic and low-nutrient environment [4]. It appears that HCC cell proliferation would be reduced in a low trophic state; on the contrary, HCC cells tolerate low-nutrient environments well and maintain their ability to proliferate rapidly. In some cases, a tumor larger than 10 cm in diameter is formed [5]. Currently, the question of how HCC cells obtain sufficient energy to maintain rapid proliferation under low nutritional status has not been answered.

Another interesting phenomenon is that autophagy is increased when solid tumors are formed in the abovementioned severe environment. The increased autophagy in solid tumors is an adaptive behavior in response to the harsh microenvironment. Autophagy is a process wherein the double membrane is shed from the rough-surface endoplasmic reticulum of the ribosomal area and forms an autophagosome, which can envelop part of the cytoplasm and cell organelle protein composition and merge with a lysosome to form an autolysosome, which eventually degrades the autophagosome contents [6]. The process yields the energy or material a cancer cell needs to survive. Many studies have shown that autophagy plays an important role in normal cell maintenance and in tumorigenesis, drug resistance, and other pathophysiological processes [7–9]. In conditions of hypoxia and low nutrition in particular, autophagy is a protective mechanism for HCC cells. Recent studies have shown that autophagy can promote HCC cell survival and maintain proliferation by influencing lipid metabolism in hypoxic environments [10]. A study on autophagy found that in the process of carcinogenesis in Ras-mediated transformation, autophagy can promote glucose uptake and utilization, and that inhibiting autophagy caused an obvious decrease in glucose uptake [11]. As autophagy is a protective process in cancer cell survival that requires much energy and material, then is glucose metabolism, the major energy delivery pathway, regulated by autophagy? How does autophagy regulate glucose metabolism?

The metabolic associated enzymes such as pyruvate kinase, hexokinase, lactic dehydrogenase (LDH), and transport proteins such as glucose transport protein and monocarboxylate transporter (MCT) are key metabolic regulators. MCTs play an important role in lactic acid transport and clear H^+^ in cancer cells [12]. In addition, recent studies have shown that MCT overexpression may play an important role in tumorigenesis. At present, the Wnt pathway can induce HCC by activating the downstream target genes such as c-*MYC*, c-*JUN*, cyclin D1 (*CCND1*), and vascular endothelial growth factor (*VEGF*) [13]. However, whether the Wnt pathway is involved in HCC metabolism is unclear.

In the present study, we explored the mechanism of autophagy in influencing glycolysis, invasion, and metastasis in HCC cells. Autophagy enhances glucose uptake and lactic acid production by upregulating MCT1 expression and the activation of Wnt/β-catenin signaling. Therefore, we investigated the mechanism of autophagy-regulated glycolysis in HCC cells. Our findings provide an experimental and theoretical basis for the further study of autophagy in HCC development and treatment.

## Methods

### Clinical samples

Eighty-five pairs of HCC tissues and the adjacent tissues were acquired from the Liaoning Cancer Hospital and Institute. The tissues were frozen in liquid nitrogen and then immediately stored at −80 °C. The hospital ethics committee approved the protocol, and all patients provided gave consent for the utilization of their tissue samples in this study.

### Cell culture and transfection

Two HCC cell lines (SMMC-7721, HepG2) were purchased from Shanghai Institute of Cell Bank (Shanghai, China) and grown in RPMI 1640 medium (BioWhittaker, Walkersville, MD, USA) supplemented with 10% fetal bovine serum (HyClone, Logan, UT, USA) at 37 °C in 5% CO_2_ and saturated moisture. For investigating the role of β-catenin in autophagy, the cells were transfected with small interfering RNAs (siRNAs) targeting three β-catenin loci (si-β-catenin-1, si-β-catenin-2, si-β-catenin-3) or scrambled negative control (si-NC) designed and synthetized by GeneChem (Shanghai, China). The β-catenin siRNA sequences were: si-β-catenin-1, 5′-GAUGGUGUCUGCUAUUGUACG-3′ (sense) and 5′-GGACAAGGAAGCUGCAGAAGC-3′ (anti-sense); si-β-catenin-2, 5′-UUCUGCAGCUUCCUUGUCCUG-3′ (sense) and 5′-UUGUGAUCCAUUCUUGUGCAU-3′ (anti-sense); si-β-catenin-3, 5′-GAUGGUGUCUGCUAUUGUACG-3′ (sense) and 5′-UACAAUAGCAGACACCAUCUG-3′ (anti-sense). For investigating the relationship between starvation-induced autophagy and glycolysis, the cells were treated with Earle’s balanced salts solution (EBSS) for 6 h or with 10 μM 3-methyladenine (3-MA, Sigma, Shanghai, China) for 6 h.

### Quantitative real-time PCR

Total cell RNA was extracted using TRIzol (Invitrogen, Life Technologies, Carlsbad, CA, USA). First-strand complementary DNA (cDNA) was synthesized using random primers using a RevertAid First Strand cDNA Synthesis Kit (Thermo Scientific, Waltham, MA, USA). SYBR Premix Ex Taq II (Takara, Dalian, China) was used to perform the quantitative real-time PCR on a CFX96 Touch Real-Time PCR Detection System (Bio-Rad, Hercules, CA, USA), and the results were normalized with U6 or β-actin as the internal control. The primer sequences used for the real-time PCR are in Additional file 1.

### Western blotting

Radioimmunoprecipitation assay buffer containing protease inhibitor (Roche, Nutley, NJ, USA) was used to lyse the cells. The protein samples were separated using sodium dodecyl sulfate–polyacrylamide gel electrophoresis and transferred to polyvinylidene difluoride membranes (Millipore, Billerica, MA, USA). The membranes were incubated with primary antibodies against MCT1, β-catenin, matrix metalloproteinase 2 (MMP2), MMP9, and β-actin (Proteintech, Wuhan, China) overnight at 4 °C. Enhanced chemiluminescence reagents (Wanlei, Shenyang, China) were used to assess protein expression; protein band intensity was quantified by densitometry and normalized to the corresponding bands for β-actin.

### Migration and invasion assays

The invasion assay was performed using a 24-well Millicell chamber containing a Matrigel-coated membrane. The migration assay was performed similarly to the invasion assay except the Millicell chamber did not contain a Matrigel-coated membrane. For both assays, the HCC cells (3*10^4^/well) were seeded in the top chamber. The bottom wells were filled with complete RPMI 1640 medium for 24 h. Cells remaining on the top side of the membrane were wiped away with cotton swabs, and cells that were on the bottom side of the membrane were fixed with methanol, stained with 0.1% crystal violet, and the cells in five random fields were counted and the average value was calculated. Each experiment was conducted in triplicate.

### Immunostaining

Paraffin sections were used for investigating MCT1 expression in human HCC tissues. Antigen retrieval was performed using citric acid; dehydration and clearing were performed using gradient alcohol. The sections were then stained using an immunohistochemical kit (ZSGB-BIO, Beijing, China) according to the manufacturer’s instructions. The sections were incubated with primary antibody against MCT1 (1:100, Proteintech, Wuhan, China), counterstained with hematoxylin, and mounted. The nuclei were stained using diaminobenzidine (Beyotime, Shanghai, China), and the slides were observed under microscopy.

### Lactate level measurement

Lactate levels were detected using a Lactate Assay Kit II (Sigma). Cell samples were combined with 4× volume lactate assay buffer to dissolve, and centrifuged at 13,000 rpm for 10 min. The pellet was discarded, and a 10-KDa overspeed centrifugal tube was used to remove the effect of LDH. The supernatant was dispensed in a 96-well plate. Lactate assay buffer (50 μL) was added to each well; empty wells were filled with 50–96 μL lactate assay buffer (total volume, 100 μL), pipette-mixed, and incubated for 30 min at room temperature away from light before the absorbance was measured at 450 nm.

### Glucose measurement

Glucose levels were detected using a High Sensitivity Glucose Assay Kit (Sigma). Cells (1 × 10^6^) were combined with 10^6^ μL glucose and assay buffer and centrifuged at 12,000 rpm for 5 min; the supernatant was collected while the pellet was discarded. Protein was removed from the samples using a 10-KDa molecular weight cut-off spin filter. Each well in a 96-well plate was filled with 25 μL sample, and glucose assay buffer was added to make up a final volume of 50 μL. Empty wells were filled with 50–96 μL glucose assay buffer (total volume, 100 μL), pipette-mixed, and incubated at 37 °C for 30 min away from light before the luminosity was measured (λex = 535/λem = 587 nm).

### Cell proliferation

Cell Counting Kit-8 (CCK-8, Beyotime) was used to detect cell proliferation. Cells (5 × 10^3^/ml) were seeded in 96-well plates, incubated in 5% CO_2_ overnight at 37 °C, and then incubated for 1 h with 10 μL CCK-8 reagent. We measured the CCK-8 absorbance at 450 nm using a microplate reader to determine the rate of cell growth.

### Immunofluorescence

The cells were cultured and seeded in 6-well plates, washed with phosphate-buffered saline, and fixed with 4% polyformaldehyde. Then, primary antibody against microtubule-associated protein light chain 3 (LC3, 1:100, Cell Signaling Technology, Massachusetts, US) or β-catenin (1:100, Cell Signaling Technology, Massachusetts, US) was added, and the plates were incubated at 4 °C overnight. After washing with PBS, fluorescein isothiocyanate–labeled secondary antibody (Bioss, Beijing, China) was added and incubated for 2 h. The cells were counterstained with diamidinophenylindole (Bioss) and visualized under a confocal microscope.

### Luciferase reporter assays

The β-catenin-mediated transcriptional activation was detected by TOPflash luciferase reporter plasmid, reporter activity was normalized to the control Renilla. The reporter assays were conducted following previously described [14].

### Transmission electron microscopy

The cells were fixed in 3% glutaraldehyde and fixed in 0.1 M sodium cacodylate buffer. Then, the cells were dehydrated in 50–100% ethanol gradient and embedded in araldite; ultrathin sections were obtained (50–60 nm) and stained with uranyl acetate and lead citrate. Images were viewed using a JEM-1400 transmission electron microscope at 80 kV.

### Statistical analysis

Data analyses were performed using SPSS 17.0 software (SPSS Inc., Chicago, IL, USA). The results are presented as the mean ± SD; statistical analyses were performed using the Student *t*-test or analysis of variance. The relationship between the expression of two genes was analyzed using Pearson correlation analysis. *P*-values <0.05 were considered significant.

## Results

### Starvation upregulated autophagy in HCC cells

**Fig. 1:**
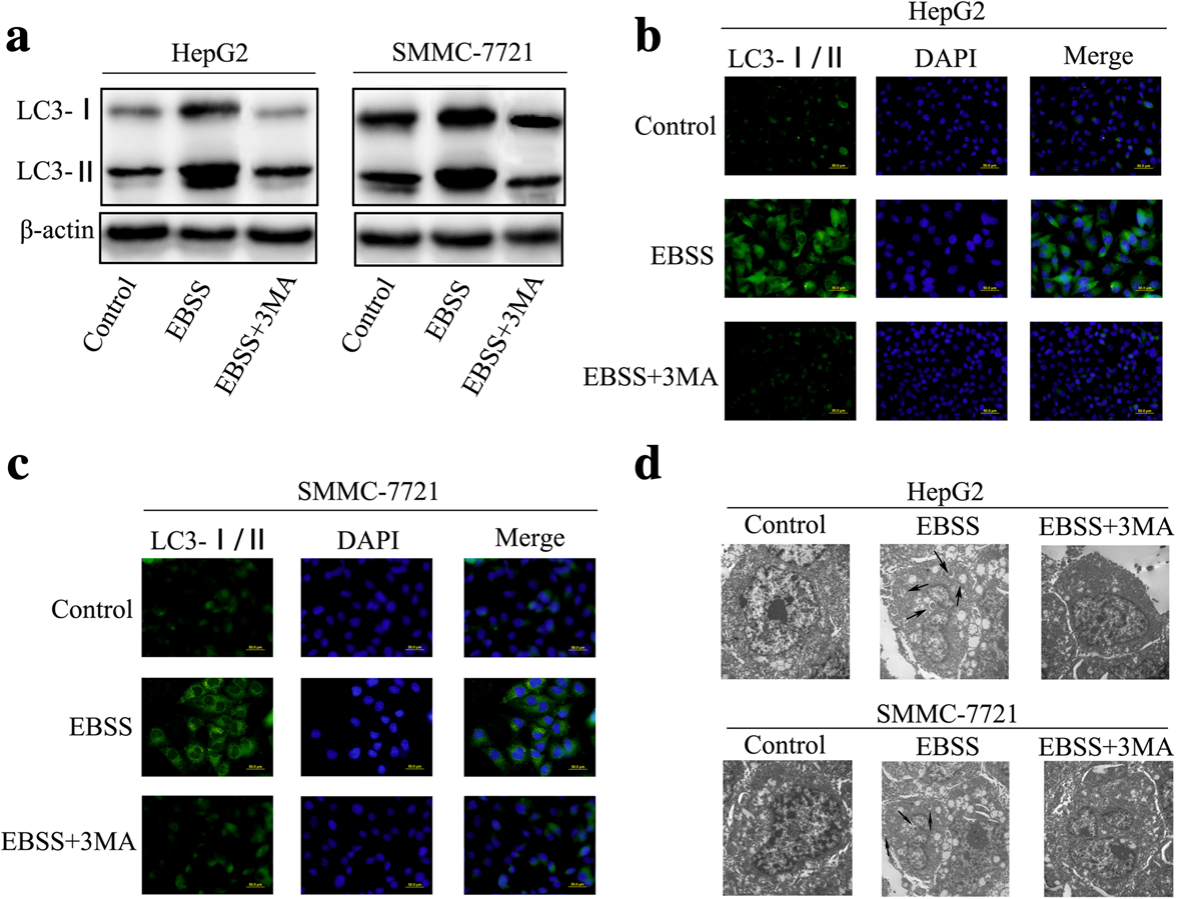
Starvation upregulates autophagy in HCC cells. (**a**) The expression levels of LC3 I and LC3 II in HepG2 cells and SMMC-7721 cells cultured with control, EBSS or EBSS + 3MA for 6 h. (**b**, **c**) Immunofluorescence showed significantly increased of LC3 protein accumulation in starved HepG2 cells and SMMC-7721 cells cultured with control, EBSS or EBSS + 3MA for 6 h. (**d**) transmission electron microscopy showed autophagosomes in the starved HepG2 and SMMC-7721 cells cultured with control, EBSS or EBSS + 3MA for 6 h. Data are shown as the mean ± SEM of three independent experiments

Starvation or nutrient deficiency can upregulate autophagy. Accordingly, we cultured HepG2 and SMMC-7721 cells with EBSS, which contains neither nutrients nor serum, for 6 h to induce autophagy. After the 6 h starvation, there was significant autophagy in the control cells compared with the cells treated with the autophagy inhibitor 3-MA. Western blotting showed that both LC3-II expression and the LC3-II/LC3-I ratio were increased after starvation (Fig. 1a). Immunofluorescence showed significantly increased LC3 protein accumulation after starvation (Fig. 1b, c). Transmission electron microscopy revealed more characteristic autophagosomes in the starved cells (Fig. 1d). These data confirm that 6 h starvation can induce autophagy in HCC cells.

### Autophagy promoted HCC cell migration and invasion

**Fig. 2:**
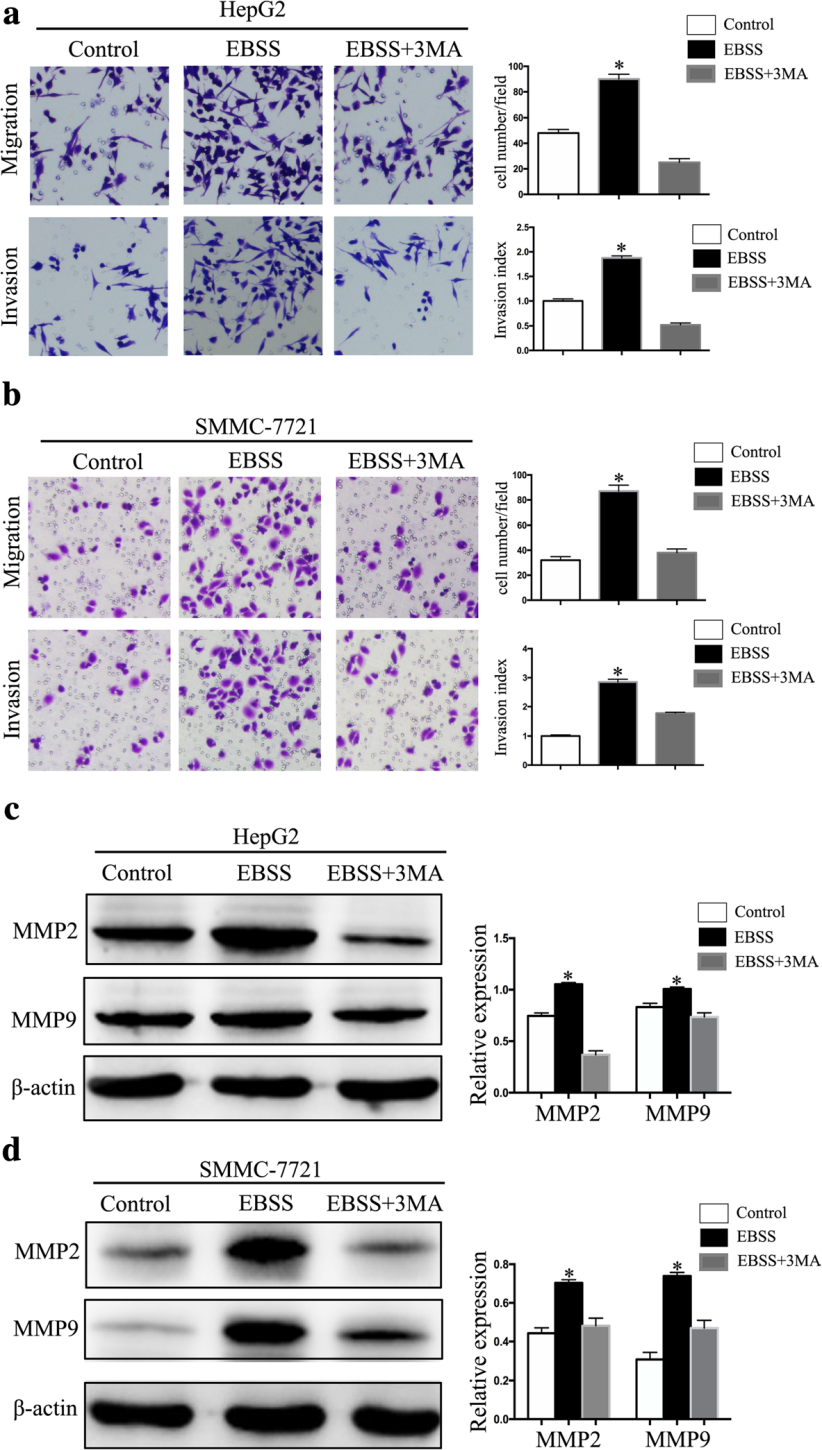
Autophagy promoted HCC cell migration and invasion. (**a**, **b**) Migration and Matrigel invasion assays were used to detect the migration and invasion of HepG2 cells and SMMC-7721 cells cultured with control, EBSS or EBSS + 3MA for 6 h. Representative images are shown (magnification: ×100). (**c**, **d**) Western blot showed the expression of MMP2 and MMP9 in the starved HepG2 and SMMC-7721 cells cultured with control, EBSS or EBSS + 3MA for 6 h. Data are shown as the mean ± SEM of three independent experiments. *p < 0.05

We detected the impact of autophagy on HCC cell invasion and migration via Transwell assays. Cells that had been starved for 6 h had significantly increased invasive and migration ability compared with the control and the cells treated with 3-MA (*p* < 0.05; Fig. 2a, b). MMP2 and MMP9 were also increased in the autophagy-induced cells (Fig. 2c, d). MMP2 and MMP9 are the members of matrix metalloproteinases. Their function is involved in ECM degradation, which is the one of the key prcocess in tumor metastasis. The secretion of MMP2 and other specific factors about invasion were found to depend on autophagy [15]. These results indicate that starvation-induced autophagy can promote HCC cell invasion and migration.

### Autophagy promoted HCC cell glycolysis by upregulating MCT1

**Fig. 3:**
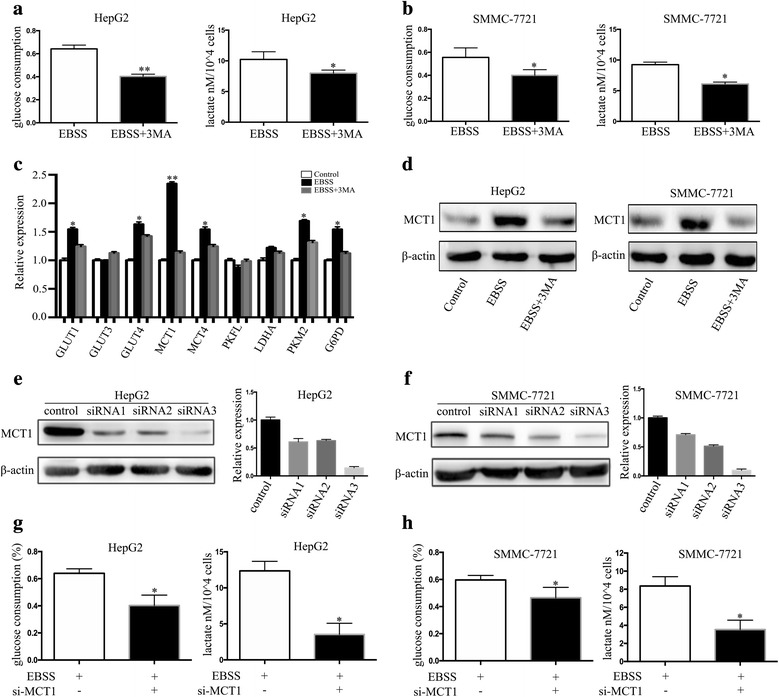
Autophagy promoted HCC cell glycolysis by upregulating MCT1. (**a**, **b**) Analysis of the consumption of glucose and production of lactate in HepG2 cells and SMMC-7721 cells cultured with control, EBSS or EBSS + 3MA for 6 h. (**c**) The expression levels of glycolysis key enzymes in the starved HepG2 and SMMC-7721 cells cultured with control, EBSS or EBSS + 3MA for 6 h were detected by real-time PCR. (**d**) Western blot showed the expression of MCT1 protein in the starved HepG2 and SMMC-7721 cells cultured with control, EBSS or EBSS + 3MA for 6 h. Data are shown as the mean ± SEM of three independent experiments. (**e**, **f**) Real-time PCR and western blot were used to examine the expression of MCT1 in HepG2 and SMMC-7721 cells transfected with MCT1 siRNA. (**g**, **h**) Analysis of the consumption of glucose and production of lactate in HepG2 cells and SMMC-7721 cells cultured with EBSS for 6 h after transfected with MCT1 siRNA . Data are shown as the mean ± SEM of three independent experiments. *p < 0.05, **p < 0.01

We examined whether autophagy can promote HCC cell glycolysis. The glucose and lactate assays showed that autophagy significantly increased HCC cell glucose consumption and lactate production rates, while inhibition of autophagy by 3-MA significantly decreased them (Fig. 3a, b). Next, we probed the expression of several key enzymes of glycolysis, and found that autophagy promoted glycolysis by upregulating MCT1 in the HCC cells. Real-time PCR used to detect the expression of glycolysis key enzymes in the HCC cells revealed significantly increased MCT1 in the starved cells; cells that had been treated with 3-MA had decreased enzyme expression (Fig. 3c), and western blotting yielded similar results (Fig. 3d). To further investigate the role of MCT1 in autophagy-induced HCC glycolysis, we detected the effect of glycolysis when MCT1 knockout in autophagy induction. The konckdown effect of three MCT1 siRNAs was performed by wetern blot. The results showed that siRNA3 could knock down the MCT1 expression effectively (Fig. 3e, f). Then we found that MCT1 knockdown decreased consumption and lactate production rates when induction of autophagy by EBSS (Fig. 3g, h), which revealed MCT1 could participate in autophagy-induced glycolysis in HCC cells.

### Autophagy activated Wnt/β-catenin signaling in HCC cells

**Fig. 4:**
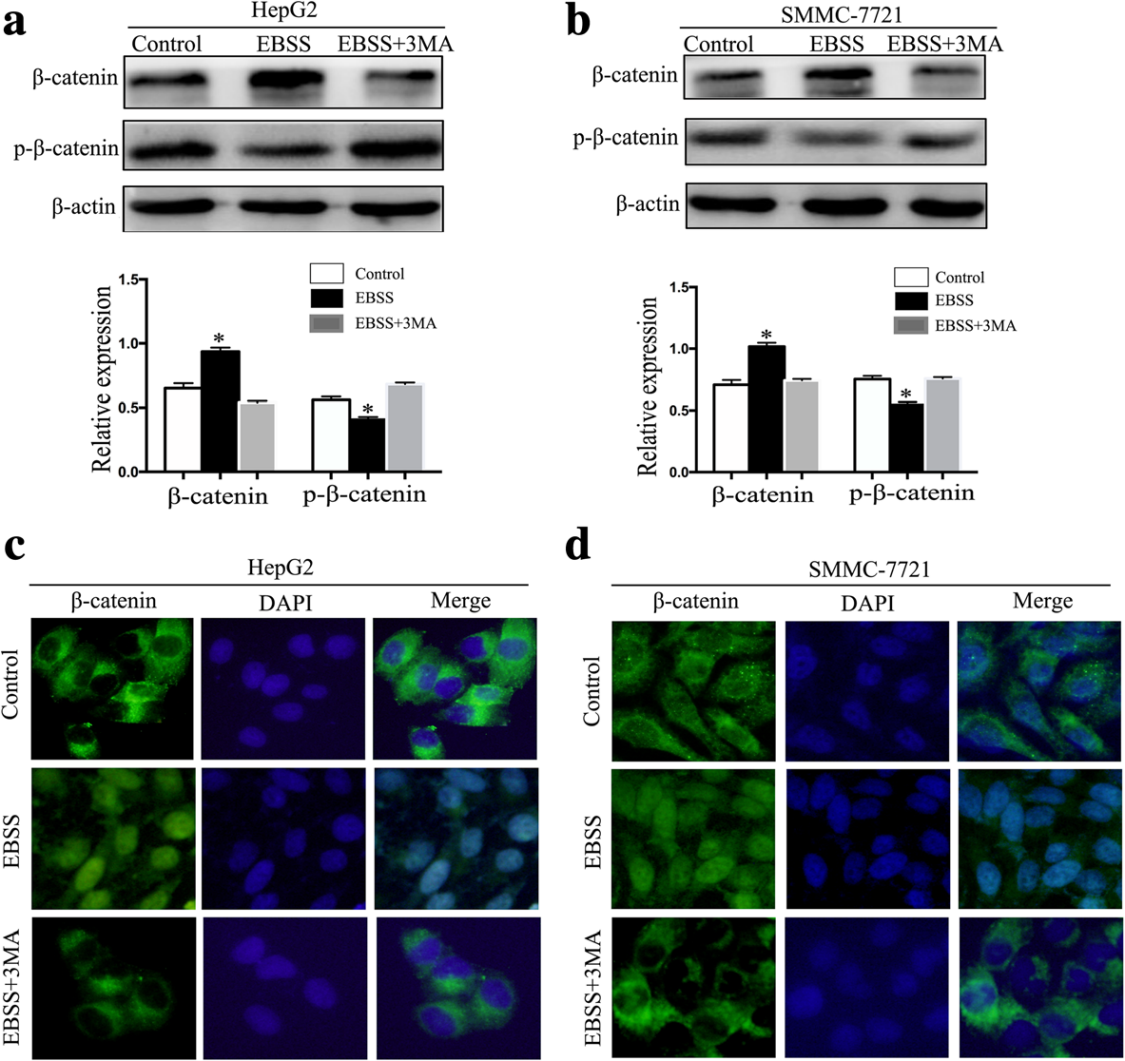
Autophagy activated Wnt/β-catenin signaling in HCC cells. (**a**, **b**) Western blot showed the expression of β-catenin and p-β-catenin in the starved HepG2 and SMMC-7721 cells cultured with control, EBSS or EBSS + 3MA for 6 h. (**c**, **d**) Immunofluorescence showed the location of β-catenin in starved HepG2 cells and SMMC-7721 cells cultured with control, EBSS or EBSS + 3MA for 6 h. Data are shown as the mean ± SEM of three independent experiments. *p < 0.05

Wnt/β-catenin signaling plays an oncogenic role in many cancers; recent research has reported that Wnt/β-catenin is also involved in autophagy in human non–small cell lung cancer cells [16]. We therefore detected whether autophagy can upregulate MCT1 via Wnt/β-catenin signaling in HCC cells. Western blotting showed increased β-catenin expression following 6 h starvation with EBSS as compared with the control, while β-catenin expression was decreased in the 3-MA–treated cells (Fig. 4a, b). Furthermore, β-catenin activity is determined by its phosphorylation status and cellular localization. We therefore examined β-catenin phosphorylation at Ser33 in HCC cells cultured with EBSS or 3-MA for 6 h. The starved cells had decreased β-catenin phosphorylation, while the autophagy-inhibited cells had increased β-catenin phosphorylation (Fig. 4a, b). Immunofluorescence examination of the cellular localization of β-catenin showed that autophagy increased β-catenin nuclear accumulation after 6 h starvation (Fig. 4c, d). These results indicate that starvation-induced autophagy can activate β-catenin.

### β-catenin downregulation inhibited autophagy-induced glycolysis in HCC cells

**Fig. 5:**
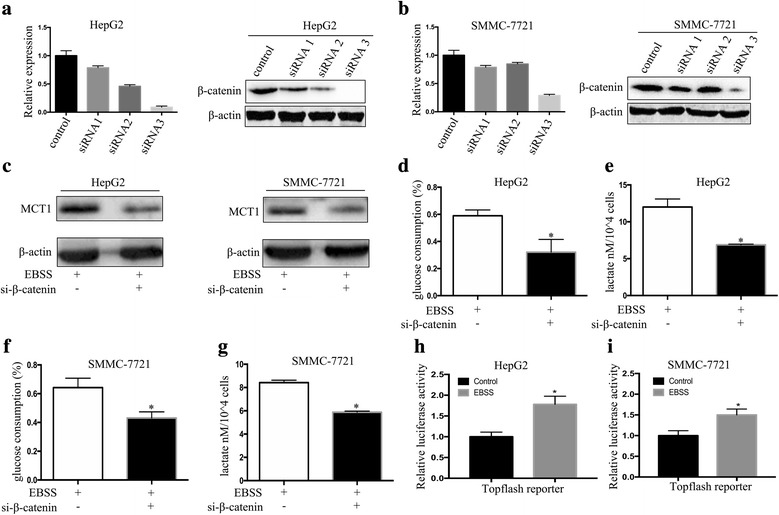
β-Catenin downregulation inhibited autophagy-induced glycolysis in HCC cells. (**a**, **b**) Real-time PCR and western blot were used to examine the expression of β-catenin in HepG2 and SMMC-7721 cells transfected with β-catenin siRNA. (**c**) The expression of MCT1 in HepG2 cells and SMMC-7721 cells cultured with EBSS for 6 h after transfected with β-catenin siRNA. (**d**-**g**) Analysis of the consumption of glucose and production of lactate in HepG2 cells and SMMC-7721 cells cultured with EBSS for 6 h after transfected with β-catenin siRNA . Data are shown as the mean ± SEM of three independent experiments. (**h**, **i**) The effect of activation of autophagy with EBSS on Wnt/β-catenin signaling pathway was detected by dual-luciferase reporter assays in HepG2 cells and SMMC-7721 cells. *p < 0.05

To further determine the role β-catenin plays in autophagy-promoted glycolysis in HCC cells, we transfected HCC cells with β-catenin siRNAs for 48 h to decrease β-catenin expression. The β-catenin levels were measured using real-time PCR and western blotting to evaluate transfection efficiency (Fig. 5a, b). We then examined the effect of β-catenin on autophagy-induced glycolysis in the HCC cells. Western blotting showed that the β-catenin knockdown decreased MCT1 expression significantly (Fig. 5c), and decreased glucose consumption and lactate production rates (Fig. 5d–g). These findings indicate that autophagy upregulates MCT1 and induces HCC cell glycolysis by activating Wnt/β-catenin signaling. Moreover, more researches indicated β-catenin was involved in the regulation of target gene expression by promoting gene transcription. To further investigate the mechanism, the Top-Luc Flash reporter and pRL-TK (negative control) were performed a special luciferase experiment. Our results indicated that induction of autophagy by EBSS increased luciferase activity significantly in both HepG2 and SMMC-7721 cells (Fig.5h, 5i). Thus indiacted that β-catenin can promote MCT1 expression by promoting transcription.

### High MCT1 expression correlated positively with LC3B in HCC tissues

**Fig. 6:**
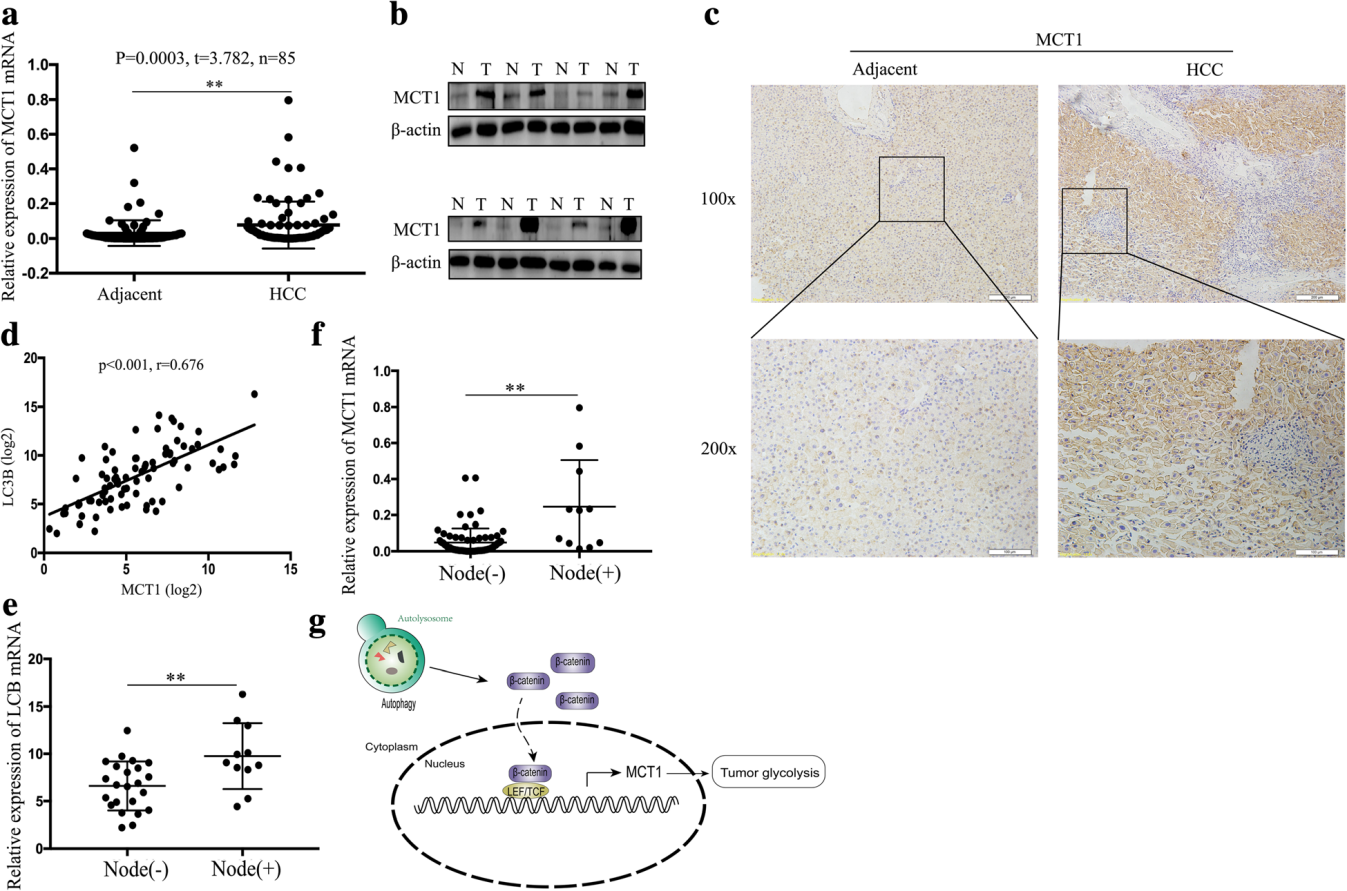
High MCT1 expression correlated positively with LC3B in HCC tissues. (**a**) The expression levels of MCT1 in 85 pairs of tumor tissues and adjacent tissues were detected by real-time PCR. (**b**) The representative expression of MCT1 levels in adjacent tissues (N) and tumor tissues (T) by Western blot. (**c**) Representative immunostaining of MCT1 in HCC tissues and adjacent tissues (magnification: ×100, ×200). (**d**) Pearson correlation to analyse the expression relationship between MCT1 mRNA and LC3B mRNA in 85 HCC tissues (*r* = 0.676, *P* < 0.001). (**e**) Relative MCT1 expression in metastatic(*n* = 11) cases versus non-metastatic cases(*n* = 74). (**f**) Correlation between autophagy marker LC3B and metastasis. (**g**) A Schematic model of autophagy promoting MCT1 expression by β-catenin to influence glycolysis in HCC. *p < 0.05, **p < 0.01

We examined MCT1 expression in 85 HCC tissues via western blotting and real-time PCR, which showed that both MCT1 protein and mRNA were highly expressed in HCC tissues compared with the adjacent tissues (Fig. 6a, b). Immunohistochemical analysis indicated that MCT1 was mainly expressed in the cell membrane in the HCC tissues, and that its expression was increased in the HCC tissues (Fig. 6c). LC3 is a marker protein in autophagy; some studies have shown that it is highly expressed in HCC tissues [17, 18]; Accordingly, we used the Pearson correlation to analyze the relationship between the expression of MCT1 mRNA and LC3B mRNA in the 85 HCC tissues, which determined that MCT1 mRNA expression correlated positively with that of LC3B mRNA (*r* = 0.676, *p* < 0.001) (Fig. 6d). Moreover, we divided the 85 tissues into 2 groups by lymph node metastases. And we we analysed the relative MCT1 expression in metastatic cases (*n* = 11) versus non-metastatic cases (*n* = 74). The results showed that MCT1 expression significantly higher in patients with lymph node metastases (Fig. 6e). Additionally, we then analysed the correlation between autophagy marker LC3B and metastasis. The results showed that LC3B expression was significantly higher in patients with lymph node metastases than without lymph node metastases (Fig. 6f). Thus revealed that autophagy is associated with metastasis in HCC.

## Discussion

Autophagy is a dynamic physiological process that can generate energy and nutrients for cell survival during stressors. From this aspect, the role of autophagy changes during the course of tumorigenesis and tumor development [19]. Due to the heterogeneity of cancer, the process is highly complex between tumor types. In HCC, autophagy can suppress tumor initiation in normal liver by maintaining intracellular homeostasis. On the contrary, autophagy can support liver cancer cell growth and proliferation when a tumor is established [20]. However, the detailed mechanisms on how autophagy influences cancer cell progression are not fully understood. In this study, we determined the role of autophagy in the energy metabolism of HCC cells.

Recent research has revealed that autophagy is of value for exploring the potential prognostic biomarkers and therapy targets in patients with HCC [21]. The ClinicalTrials.gov website lists over 50 ongoing randomized controlled trials on the role of autophagy in cancer therapy. One trial is evaluating whether sorafenib/hydroxychloroquine will have improved efficacy when compared to sorafenib alone and in patients progressing to sorafenib, and if the addition of hydroxychloroquine would lead to disease stability in patients with advanced HCC (Trial registration ID: NCT03037437). In a previous study, we focused on the autophagy regulators and found that the long noncoding RNA HOTAIR participates in autophagy promotion in HCC cells [22]. However, the downstream effect of autophagy in cancer cells is unclear, and its elucidation is important for exploiting autophagy as a therapeutic strategy.

The role of autophagy in tumor progression has been explored recently. An increasing number of studies has indicated that autophagy can help tumor cells adapt to the tumor environment and survive stressors. Several studies have found that autophagy can regulate the migration and invasive ability of cancer cells. Galavotti et al. demonstrated that autophagy can promote glioblastoma stem cell migration and invasion via the autophagy-associated factors DRAM1 and p62 [23]. Indelicato et al. showed that, in MDA-MB-231 cells, autophagy can regulate cell invasion by altering the cytoskeleton [24]. To explore the role of autophagy in HCC cell invasion, we established a HCC cell model in which autophagy was induced or inhibited. Then, we detected the effect of autophagy on cell migration and invasion. The results in the two HCC cell lines (HepG2 and SMMC-7721) showed that autophagy can promote HCC cell invasion. We also assessed the expression of MMP2 and MMP9, enzymes that can catalyze collagen hydrolysis. MMP2 and MMP9 were upregulated when autophagy was activated in the HCC cells. Our work indicates that defective autophagy may be a therapeutic intervention target for preventing HCC cell invasion.

The metabolism model of cancer cells differs from that of normal cells. Cancer cells mainly depend on aerobic glycolysis to provide energy and maintain cellular homeostasis, which is critical for cancer cell proliferation and progression; this is termed the Warburg effect. Generally, autophagy is considered a process that can provide energy and components for surviving stress. However, the connection between autophagy and metabolism is unclear. Here, we investigated the relationship between autophagy and glycolysis in HCC cells. We found that activating autophagy in HCC cells increased glucose consumption and lactate production; on the contrary, inhibiting autophagy led to decreased glucose consumption and lactate production. This indicates that autophagy can promote glycolysis in HCC cells. Accumulating evidence indicates that cellular metabolism and autophagy are related, but the molecular link is unclear. To probe the expression of the metabolism-associated genes, we detected several metabolism enzymes in HCC cells using real-time PCR. We detected an obvious increase in MCT1, which was reversed by the autophagy inhibitor 3-MA. Then, we detected MCT1 protein expression under the same treatment conditions to confirm the role of MCT1 in autophagy-induced glycolysis, and found that autophagy can lead to increased MCT1 protein expression as well. These results indicate that MCT1 may be involved in autophagy-induced glycolysis. MCT1 mainly transports pyruvate, while MCT4 primarily transports lactate [25–28].

Next, we explored the mechanism by which autophagy promotes MCT1 expression by activating Wnt/β-catenin signaling. β-Catenin is an important second messenger of classic Wnt signaling. Under normal physiological conditions, β-catenin binds with glycogen synthase kinase 3 (GSK3), axin, adenomatous polyposis coli, and Dishevelled (DSH). β-Catenin can be phosphorylated by GSK3β and is eventually degraded. Once Wnt signaling is activated, it is difficult to phosphorylate and degrade β-catenin; therefore, free β-catenin accumulates. Eventually, the increased β-catenin is transported to the nucleus and leads to the increase or decrease in the expression of specific genes. Much research has shown that altered Wnt signaling is involved in HCC occurrence and development [29–31]. Recently, it was reported that Wnt signaling activation can increase colon cancer cell glycolysis and promote colon cancer cell proliferation [32]. Further research shows that it relies on Wnt activation of the downstream target pyruvate dehydrogenase kinase 1 (PDK1) [33]. Another study showed that β-catenin can regulate metabolic reprogramming via significant modulation of lipogenic enzyme expression and activity [34]. These findings indicate that Wnt/β-catenin signaling plays a key role in cancer cell metabolism. In this study, we determined that Wnt/β-catenin is involved in autophagy-induced glycolysis.

## Conclusions

Overall, autophagy activation promotes metastasis and glycolysis in HCC cells. Autophagy induces MCT1 expression by activating Wnt/β-catenin signaling. This study makes the connection between autophagy and glucose metabolism in HCC cells and may provide a potential therapeutic target for HCC.

## Additional file

Additional file 1: Primers used for Quantitative real-time PCR. (DOCX 67 kb)

## Abbreviations

3-MA: 3-Methyladenine
CCK-8: Cell Counting Kit-8
CCND1: Cyclin D1
cDNA: Complementary DNA
DSH: Dishevelled
EBSS: Earle’s balanced salts solution
GSK3: Glycogen synthase kinase 3
HCC: Hepatocellular carcinoma
LC3: Microtubule-associated protein light chain 3
LDH: Lactic dehydrogenase
MCT1: Monocarboxylate transporter 1
MMP2: Matrix metalloproteinase 2
PDK1: Pyruvate dehydrogenase kinase 1;
real-time PCR: Quantitative reverse transcription–PCR
si-NC: Scrambled negative control
siRNA: Small interfering RNA
VEGF: Vascular endothelial growth factor

## References

[1] Ingle PV, Samsudin SZ, Chan PQ, Ng MK, Heng LX, Yap SC, Chai AS, Wong AS (2016). Development and novel therapeutics in hepatocellular carcinoma: a review. Ther Clin Risk Manag.

[2] Kee HJ, Cheong JH (2014). Tumor bioenergetics: an emerging avenue for cancer metabolism targeted therapy. BMB Rep.

[3] Beyoglu D, Imbeaud S, Maurhofer O, Bioulac-Sage P, Zucman-Rossi J, Dufour JF, Idle JR (2013). Tissue metabolomics of hepatocellular carcinoma: tumor energy metabolism and the role of transcriptomic classification. Hepatology.

[4] Schlachterman A, Craft WW, Hilgenfeldt E, Mitra A, Cabrera R (2015). Current and future treatments for hepatocellular carcinoma. World J Gastroenterol.

[5] Li L, Wang H (2016). Heterogeneity of liver cancer and personalized therapy. Cancer Lett.

[6] Jiang P, Mizushima N (2014). Autophagy and human diseases. Cell Res.

[7] Ghavami S, Gupta S, Ambrose E, Hnatowich M, Freed DH, Dixon IM (2014). Autophagy and heart disease: implications for cardiac ischemia-reperfusion damage. Curr Mol Med.

[8] Hashimoto D, Blauer M, Hirota M, Ikonen NH, Sand J, Laukkarinen J (2014). Autophagy is needed for the growth of pancreatic adenocarcinoma and has a cytoprotective effect against anticancer drugs. Eur J Cancer.

[9] Ryter SW, Choi AM (2015). Autophagy in lung disease pathogenesis and therapeutics. Redox Biol.

[10] Toshima T, Shirabe K, Matsumoto Y, Yoshiya S, Ikegami T, Yoshizumi T, Soejima Y, Ikeda T, Maehara Y (2014). Autophagy enhances hepatocellular carcinoma progression by activation of mitochondrial beta-oxidation. J Gastroenterol.

[11] Lock R, Roy S, Kenific CM, JS S, Salas E, Ronen SM, Debnath J (2011). Autophagy facilitates glycolysis during Ras-mediated oncogenic transformation. Mol Biol Cell.

[12] Pedersen SF, Kramhoft B, Jorgensen NK, Hoffmann EK (1996). Shrinkage-induced activation of the Na+/H+ exchanger in Ehrlich ascites tumor cells: mechanisms involved in the activation and a role for the exchanger in cell volume regulation. J Membr Biol.

[13] Moeini A, Cornella H, Villanueva A (2012). Emerging signaling pathways in hepatocellular carcinoma. Liver Cancer.

[14] Wickstrom M, Dyberg C, Milosevic J, Einvik C, Calero R, Sveinbjornsson B, Sanden E, Darabi A, Siesjo P, Kool M (2015). Wnt/beta-catenin pathway regulates MGMT gene expression in cancer and inhibition of Wnt signalling prevents chemoresistance. Nat Commun.

[15] Lock R, Kenific CM, Leidal AM, Salas E, Debnath J (2014). Autophagy-dependent production of secreted factors facilitates oncogenic RAS-driven invasion. Cancer Discov.

[16] Peng Y, Cao J, Yao XY, Wang JX, Zhong MZ, Gan PP, Li JH (2017). TUSC3 induces autophagy in human non-small cell lung cancer cells through Wnt/beta-catenin signaling. Oncotarget.

[17] Lee YJ, Hah YJ, Kang YN, Kang KJ, Hwang JS, Chung WJ, Cho KB, Park KS, Kim ES, Seo HY (2013). The autophagy-related marker LC3 can predict prognosis in human hepatocellular carcinoma. PLoS One.

[18] WY W, Kim H, Zhang CL, Meng XL, ZS W (2014). Clinical significance of autophagic protein LC3 levels and its correlation with XIAP expression in hepatocellular carcinoma. Med Oncol.

[19] Levy JMM, Towers CG, Thorburn A. Targeting autophagy in cancer. Nat Rev Cancer. 2017; 10.1038/nrc.2017.53 PMC5975367 28751651

[20] White E (2015). The role for autophagy in cancer. J Clin Invest.

[21] Liu L, Liao JZ, He XX, Li PY. The role of autophagy in hepatocellular carcinoma: friend or foe. Oncotarget. 2017; 10.18632/oncotarget.17202 PMC5593678 28915706

[22] Yang L, Zhang X, Li H, Liu J (2016). The long noncoding RNA HOTAIR activates autophagy by upregulating ATG3 and ATG7 in hepatocellular carcinoma. Mol BioSyst.

[23] Galavotti S, Bartesaghi S, Faccenda D, Shaked-Rabi M, Sanzone S, McEvoy A, Dinsdale D, Condorelli F, Brandner S, Campanella M (2013). The autophagy-associated factors DRAM1 and p62 regulate cell migration and invasion in glioblastoma stem cells. Oncogene.

[24] Indelicato M, Pucci B, Schito L, Reali V, Aventaggiato M, Mazzarino MC, Stivala F, Fini M, Russo MA, Tafani M (2010). Role of hypoxia and autophagy in MDA-MB-231 invasiveness. J Cell Physiol.

[25] Dovmark TH, Saccomano M, Hulikova A, Alves F, Swietach P (2017). Connexin-43 channels are a pathway for discharging lactate from glycolytic pancreatic ductal adenocarcinoma cells. Oncogene.

[26] Hong CS, Graham NA, Gu W, Espindola Camacho C, Mah V, Maresh EL, Alavi M, Bagryanova L, Krotee PA, Gardner BK (2016). MCT1 modulates cancer cell pyruvate export and growth of tumors that co-express MCT1 and MCT4. Cell Rep.

[27] Tseng HY, Chen YA, Jen J, Shen PC, Chen LM, Lin TD, Wang YC, Hsu HL (2017). Oncogenic MCT-1 activation promotes YY1-EGFR-MnSOD signaling and tumor progression. Oncogene.

[28] Vallee A, Lecarpentier Y, Guillevin R, Vallee JN (2017). Aerobic glycolysis hypothesis through WNT/Beta-catenin pathway in exudative age-related macular degeneration. J Mol Neurosci.

[29] Sakurai Y, Kubota N, Takamoto I, Obata A, Iwamoto M, Hayashi T, Aihara M, Kubota T, Nishihara H, Kadowaki T (2017). Role of insulin receptor substrates in the progression of hepatocellular carcinoma. Sci Rep.

[30] Yen CH, Lai CC, Shia TH, Chen M, HC Y, Liu YP, Chang FR. Gynura Divaricata attenuates tumor growth and tumor relapse after cisplatin therapy in HCC xenograft model through suppression of cancer stem cell growth and Wnt/beta-catenin signalling. J Ethnopharmacol. 2018;213:366-75. 10.1016/j.jep.2017.07.019 28729225

[31] Zhang J, Lai W, Li Q, Yu Y, Jin J, Guo W, Zhou X, Liu X, Wang Y (2017). A novel oncolytic adenovirus targeting Wnt signaling effectively inhibits cancer-stem like cell growth via metastasis, apoptosis and autophagy in HCC models. Biochem Biophys Res Commun.

[32] Pate KT, Stringari C, Sprowl-Tanio S, Wang K, TeSlaa T, Hoverter NP, McQuade MM, Garner C, Digman MA, Teitell MA (2014). Wnt signaling directs a metabolic program of glycolysis and angiogenesis in colon cancer. EMBO J.

[33] Lee M, Chen GT, Puttock E, Wang K, Edwards RA, Waterman ML, Lowengrub J (2017). Mathematical modeling links Wnt signaling to emergent patterns of metabolism in colon cancer. Mol Syst Biol.

[34] Vergara D, Stanca E, Guerra F, Priore P, Gaballo A, Franck J, Simeone P, Trerotola M, De Domenico S, Fournier I (2017). Beta-catenin knockdown affects mitochondrial biogenesis and lipid metabolism in breast cancer cells. Front Physiol.

