# Supplementary material for: Autophagy promotes metastasis and glycolysis by upregulating MCT1 expression and Wnt/β-catenin signaling pathway activation in hepatocellular carcinoma cells
Source: J Exp Clin Cancer Res. 2018 Jan 19;37:9. doi: 10.1186/s13046-018-0673-y (PMC5775607; doi:10.1186/s13046-018-0673-y)
Supplement: Additional file 1: — Primers used for Quantitative real-time PCR. (DOCX 67 kb) [file 13046_2018_673_MOESM1_ESM.docx]

Primers used for Quantitative real-time PCR

GLUT1-F: 5′-CTTTGTGGCCTTCTTTGAAGT-3′

GLUT1-R: 5′-CCACACAGTTGCTCCACAT-3′

GLUT3-F: 5′- ATGCCCTACCAATATCCAGCA -3′

GLUT3-R: 5′- GCTCCCAGTGGACTCATCTG -3′

GLUT4-F: 5′- TGCTCGATTATGCACTGGAAGT -3′

GLUT4-R: 5′- ATGAACCCCATACTCCTTCCCAG -3′

MCT1-F:5′-AAAGTGGTGAGCTGCGACGTGA-3′

MCT1-R:5′-CGTTATATGCGCGGATCGCAG-3′

MCT4 -F:5′- GATATGGGCGCTTACCATTTTCG -3′

MCT4-R:5′- TGTGCTGCGTGACATTCCAA -3′

PFKL -F: 5’-AGATGCGCACCAGCATCAACG-3’

PFKL - R: 5’-GAACCCGGCACATTGTTGGA-3’

LDHA -F: 5′-GGAGGACCCAGCAATTAGTCT-3′

LDHA -R: 5′-GTTCACCCATCGCGGTTTAT-3′

PKM2-F:5’-ATGGCTGACACATTCCTGGAGC-3’

PKM2- R: 5’-CCTTCAACGTCTCCACTGATCG-3’

β-actin-F:5'-GGGAAATCGTGCGTGACATTAAG-3'

β-actin-R:5'-TGTGTTGGCGTACAGGTCTTTG-3'
